# Regulatory T Cells Secrete IL10 to Suppress Neuroinflammation in Early Stage after Subarachnoid Hemorrhage

**DOI:** 10.3390/medicina59071317

**Published:** 2023-07-17

**Authors:** Jingyi Zhou, Fan Yang, Huaming Li, Penglei Xu, Zefeng Wang, Fangjie Shao, Anwen Shao, Jianmin Zhang

**Affiliations:** 1 Department of Neurosurgery, Second Affiliated Hospital, School of Medicine, Zhejiang University, Hangzhou 310058, China; 2 Clinical Research Center for Neurological Diseases of Zhejiang Province, Hangzhou 310006, China; 3 Brain Research Institute, Zhejiang University, Hangzhou 310058, China; 4 Collaborative Innovation Center for Brain Science, Zhejiang University, Hangzhou 310058, China

**Keywords:** subarachnoid hemorrhage, regulatory T cell, neuroprotection, IL10, neuroinflammation

## Abstract

*Objective*: Accumulating evidence supports neuroprotective effects of regulatory T cells (Tregs) in response to brain injury. However, the precise mechanisms underlying the beneficial effects of Tregs on suppressing neuroinflammation after subarachnoid hemorrhage (SAH) remain unclear. *Methods*: We performed flow cytometry to detect the infiltration of Tregs into the brain at different time points after SAH. Behavioral tests, including Adhesive and Rotarod, were performed to assess neurological deficits in mice after SAH. Bulk RNA sequencing was used to investigate the transcriptomic change of Tregs infiltrating into the brain after SAH. qPCR was performed to verify the variation of inflammatory cytokines expression in the brain after Tregs exogenous infusion. FoxP3-DTR mice and Il10 gene KO mice were used to explore the mechanism of Tregs inhibiting neuron apoptosis after infiltrating the brain following SAH onset. *Results*: Peripheral Tregs infiltrated into the brain one day after SAH and gradually accumulated in the hemorrhagic hemisphere. An exogenous infusion of Tregs significantly improved the neurological function of mice after SAH, while poor recovery of neurological function was observed in Tregs depletion mice. Transcriptome sequencing data suggested that the immunosuppressive function of brain-infiltrated Tregs was significantly upregulated. qPCR showed that the expression of pro-inflammatory cytokines decreased in the brain of SAH mice after exogenous Tregs infusion. Bioinformatic analysis revealed that IL-10 and other cytokines secreted by brain-infiltrated Tregs were upregulated after SAH. Moreover, exogenous infusion of Il10 gene KO Tregs did not totally improve neurological function in SAH mice. *Conclusions*: Tregs infiltrated into the brain in the early stage after SAH and exerted neuroprotective effect by secreting IL-10 to suppress neuroinflammation and reduce neuron apoptosis.

## 1. Introduction

Subarachnoid hemorrhage (SAH) is a severe type of hemorrhagic stroke with high mortality and disability rates [1]. Patients with SAH have a 50% mortality rate within 30 days of onset [2], and nearly half of the survivors suffer from severe neurological dysfunction [1]. Furthermore, SAH predominates in younger patients, placing a huge burden on families and society [1]. CD4 regulatory T cells (Tregs) are a subset of T lymphocytes that highly express CD4, CD25, and the transcription factor Foxp3 [3]. These cells can suppress inflammatory response and maintain immune homeostasis [4]. Previous studies have reported that Tregs can reduce infarction volume, and improve long-term neurological function by protecting the blood–brain barrier after ischemic stroke [5,6,7]. Moreover, it has also been reported that exogenous administration of Tregs can significantly reduce brain edema, increase cerebral blood flow perfusion, and suppress intracerebral inflammatory response in mice after SAH [8]. However, the mechanism that is underlying the protective effect of Tregs in SAH remains unclear. In this study, we used flow cytometry to detect the infiltration of Tregs into the brain at different time points after SAH. Bulk RNA sequencing was used to investigate the transcriptomic change of Tregs infiltrating into the brain after SAH. Furthermore, FoxP3-DTR mice [9] and Interleukin 10 gene Knockout (Il10 gene KO) mice [10] were used to explore the mechanism of Tregs inhibiting neuron apoptosis after infiltrating into the brain following SAH onset.

## 2. Materials and Methods

### 2.1. Experiment Animals

Young male Wild Type (WT) C57BL/6 mice (25–30 g weight, 9–10 weeks old) purchased from SLAC Laboratory Animal Company Limited (Shanghai, China), young male FoxP3-DTR mice (9–10 weeks old) and young male Il10 gene KO mice (9–10 weeks old) purchased from Shanghai Model Organisms Center, Inc. (Shanghai, China) were used to induce SAH. As for Tregs depletion, Diphtheria toxin (DT, intraperitoneal, 0.05 mg/g body weight) was injected 3 days prior to SAH to deplete Tregs, and repeated every 3 days to maintain Tregs depletion until mice sacrifice [11]. As for Tregs exogenous administration, Tregs were obtained from mice spleen by using a mouse Treg cell isolation kit, according to the manufacturer’s instructions. A total of 2 × 10^6^ Tregs or equivalent volume of PBS were infused into mice through the tail vein 2 h after SAH onset [6]. While WT and Foxp3-DTR mice were housed under specific pathogen free (SPF) conditions, Il10 gene KO mice were maintained at high-health status conditions (high SPF) to avoid phenotypes change due to the husbandry environment [12]. All animals were housed in a temperature and humidity-controlled facility with a 12 h light/dark cycle. Food and water were available ad libitum. Animals were randomly assigned to sham or SAH groups and received randomized treatments. All of the animal protocol was approved by the Institutional Ethics Committee of the Second Affiliated Hospital, Zhejiang University School of Medicine (Approval code: 2022-126, Approval date: 29 August 2022). The procedures were conducted according to the National Institutes of Health’s Guide for the Care and the Use of Laboratory Animals and the ARRIVE (Animal Research: Reporting in vivo Experiments) guidelines. All efforts were made to minimize animal suffering and the number of animals used.

### 2.2. Reagents and Instruments

The antibodies used in this study include: anti-Mouse Foxp3 PE, anti-Mouse CD25 BV605, anti-Mouse CD45 Pacific Blue, anti-Mouse CD4 APC-cy7, anti-Mouse CD3e FITC, anti-Mouse CD3e PE-cy7, and were all purchased from BD Bioscience (Becton Drive Franklin Lakes, NJ, USA); The reagents used in this study mainly include: 10X RBC Lysis Buffer (00-4300-54), neural tissue isolation kit-(NTDK), Treg cell isolation kit (130-091-041) was purchased from Miltenyi (Bergisch Gladbach, Germany); Foxp3/Transcription Factor Staining Buffer Set kit (00-5523-00) was purchased from BD Bioscience (Becton Drive Franklin Lakes, NJ, USA); DT(D0564) was purchased from Sigma Aldrich (Burlington, MA, USA); PBS buffer (BL302A) was purchased from Biosharp Company (Shanghai, China); OCT embedding agent (4583), Percoll cell separation solution (P8370) and Ficoll lymphocyte separation solution (P4350) were purchased from Solarbio Company (Beijing, China); TUNEL kit (C1088), DAPI staining solution (C1006), sodium citrate antigen restoration solution (P0081), and 4% paraformaldehyde (P0099-500 mL) were purchased from Biyuntian Company (Shanghai, China); sodium pentobarbital was purchased from Merk Company (Rahway, NJ, USA). The instruments used in this study mainly include: Surgical instruments were purchased from RWD Company (Shenzhen, China); binocular stereo microscope was purchased from Bresser Company (Rhede, Germany); Velocity 18R desktop centrifuge was purchased from Dynamica Company (Victoria, Australia); CytoFLEXLX flow cytometry analyzer was purchased from Beckman Coulter Company (Brea, CA, USA); BD Aria SORP flow cytometer was purchased from BD bioscience Company (Becton Drive Franklin Lakes, NJ, USA); SP8 laser confocal microscope and CM3050S cryostat were purchased from Leica Company (Berlin, Germany).

### 2.3. SAH Model

The endovascular perforation model of SAH was performed as previously described [13]. Mice weighed 25 g were chosen, after anesthesia, the head and limbs were fixed, the skin of the mouse was cut at the middle of the neck, and the left neck muscle and connective tissue were carefully bluntly separated. While the carotid triangle was visible, the common carotid artery, external carotid artery and internal carotid artery were carefully separated. The distal end of the external carotid artery was ligated and scalded with an electronic coagulation, and hemostatic clips were placed at the common carotid and internal carotid arteries. A small hole was cut at the free end of the external carotid artery, a thread plug was inserted into the external carotid artery through the small hole and adjusted the direction to enter the internal carotid artery, then the thread plug was inserted about 10 mm, and advanced 1–2 mm after feeling the breakthrough to destroy the middle cerebral artery, while SAH was induced, withdrawn the suture, ligated external artery stump, loosen two hemostatic clips to restore brain blood flow, and finally sutured the neck skin. During the operation, the body temperature of the mice was maintained at 37 °C, and the mice were placed on an incubator until they woke up from anesthesia. In the sham group, the middle cerebral artery was not punctured, and other operations were the same.

### 2.4. SAH Severity Assessment

In order to maintain the consistency of SAH severity, we used the previously reported SAH quantitative assessment method with appropriate modifications [14]: After obtaining the mouse brain tissue, the basal surface was observed and divided into 6 areas, each area was scored according to the amount of bleeding (0–3 points), 3 points: a large number of blood clots attached to the brain tissue can be seen and the circle of Willis blood vessels cannot be identified, 2 points: moderate bleeding, the circle of Willis blood vessels can be identified, 1 point: slight bleeding, 0 point: no bleeding. The scores of the 6 areas were added together to form the SAH severity score, with a total score of 0–18 points, and the greater the score, the more severe the SAH was.

### 2.5. Gracia’s Score Test

The Garcia’s score test was performed as previously described [15]. Six tests were performed: Spontaneous activity, Symmetry in four limb movement, Forepaw outstretching, Climbing, Body proprioception and Response to vibrissae touch. Each test was scored by a 3 points system according to the following criteria: 0, severe deficits; 1, moderate deficits; 2, mild deficits; 3, no deficits. The total score of the modified Garcia Score is 18 points, which ranges from 0 (serious injury) to 18 (no injury).

### 2.6. Brain Water Content Assessment

The wet-dry method was used to assess the brain edema 24 h after SAH. Briefly, the mice underwent euthanasia and the brains were quickly collected, which were then separated into right hemisphere, left hemisphere, cerebellum, and brain stem. Afterwards, these four parts of the brain were weighed separately (wet weight) and then put into an oven at a temperature of 105 °C for three days. Dry brains were weighed (dry weight) and the brain water content was calculated as follows: [(wet weight − dry weight)/(wet weight)] × 100% [16].

### 2.7. TUNEL Staining

TUNEL staining was used to detect neuron apoptosis after SAH in mice. The tissue was soaked in 4% paraformaldehyde for one day to fix the tissue block, and then taken out and placed in 30% sucrose solution for 72 h, after sinking to the bottom, the tissue was taken out, blotted dry, embedded, and then frozen for sectioning. Permeabilization and blocking was carried out, and the brain sections were incubated in an appropriate amount of TUNEL reaction buffer in the dark at 37 °C for 1 h, then incubated with DAPI for 5 min, and finally sealed the brain slices with the mounting solution. The slices were observed using a confocal microscope. The total number of neurons and the number of TUNEL-positive neurons were counted, and the apoptosis rate of neurons was calculated as follows: the number of TUNEL-positive neurons/total number of neurons × 100%.

### 2.8. Flow Cytometry

For mouse brain tissue, the mice were anesthetized with pentobarbital, perfused with pre-cooled PBS, quickly decapitated to obtain the brain tissue, and a razor was used to cut the left hemisphere of the mouse brain which was minced moderately. The NTDK reagent was used to dissociate the minced brain tissue into a single cell suspension according to the manufacturer’s instructions. Then, the single cell suspension was filtered through a 70 um filter, and intracerebral immune cells were obtained by 30–70% Percoll gradient centrifugation, which were then incubated with extracellular antibodies for 30 min for flowcytometry analysis. For mouse blood, after using pentobarbital to anesthetize the mouse, the chest cavity was exposed, about 0.8 mL of mouse blood was extracted from the right ventricle, and peripheral blood mononuclear cells were obtained by Ficoll gradient centrifugation and 1X RBC Lysis Buffer lysis was used to remove red blood cells, followed by incubating extracellular antibodies for 30 min for flowcytometry analysis.

For Tregs staining, Foxp3/Transcription Factor Staining Buffer Set Kit was used to permeabilize the cell membrane, incubated with intracellular antibodies for 30 min, and then performed flowcytometry analysis.

### 2.9. Behavioral Tests

In this study, behavioral tests were used to assess the deficits in neurological functions (motor, sensory, learning and memory, etc.) of mice after SAH [17,18]. (1) Adhesive test: A sticky sticker with a diameter of 5 mm was pasted on the right wrist of the mouse, and the time when the mouse perceived the sticker and removed the sticker was recorded, respectively, which can be used to evaluate the sensory ability and motor ability of the mouse limbs. (2) Rotarod: before SAH modeling, the mice were trained to run on the rolling wheel until all mice stabilized at the same level. After the SAH model was established, the mice were placed on the wheel, and the speed was gradually increased from 4 rpm to 40 rpm within 5 min, and the time when the mouse fell from the wheel was recorded, which was used to reflect the limbs of the mouse’s motor function.

### 2.10. Bulk RNA Sequencing and Analysis

Single cell suspensions were isolated from brain or blood of Foxp3-DTR mice in SAH 3d or sham group, as described for the flow cytometry aforementioned. Single cells suspension was incubated with CD45-Pacific Blue antibody, CD3-PE-Cy7 antibody, CD4 APC-Cy7 antibody, CD25-BV605 antibody for 30 min on ice in the dark. As Tregs in Foxp3-DTR mice were GFP labeling, the CD45^+^CD3^+^CD4^+^CD25^+^ GFP^+^ cells in blood and CD45^high^CD4^+^CD25^+^GFP^+^ cells in the brain were sorted by FACSAriaIII (BD Bioscience). Isolated cells were lysed to RNA with Trizol and then proceeded to Bulk RNA sequencing library preparation. After the data passed quality control, R packages edgeR [19], GOplot [20] and other packages were used to downstream analysis to visualize data.

### 2.11. Real Time Fluorescence Quantitative PCR (qPCR)

Total RNA was isolated from brains (SAH hemisphere or sham brain) with the RNeasy Lipid Tissue Mini kit (74804, QIAGEN, Shanghai, China). RNA (1 μg) was used to synthesize the first strand of cDNA using the Super- script First-Strand Synthesis System for RT-PCR (11752, Invitrogen, Waltham, CA, USA), according to the manufacturer’s protocols. The program for reverse transcription was 25 °C for 10 min, 50 °C for 30 min, 85 °C for 5 min, and 4 °C maintain. PCR was performed on the Opticon 2 Real-Time PCR Detection System (Bio-Rad, Hercules, CA, USA) using corresponding primers (Table 1) and SYBR Green PCR Master Mix (330503, QIAGEN). The program for real-time PCR was 95 °C for 15 min, (94 °C for 20 s, 59 °C for 30 s, 72 °C for 30 s) for 40 cycles, melting curve from 50 °C to 92 °C, read every 0.2 °C, held for 2 s, and incubated at 8 °C. The cycle time values of each target were normalized to that of Gapdh in the same sample as an internal control. The expression levels of the mRNAs were reported as fold change versus sham brain.

### 2.12. Hematoxylin-Eosin (HE) Staining

Before HE staining, the frozen brain slices were fixed in formaldehyde for 30 min. The slices were later dyed with hematoxylin for 5 min followed by counterstaining with eosin for 3 min. The stained sections were processed sequentially through 95% alcohol, absolute ethanol, xylene and resinous mounting medium. Thereafter, these sections were observed under a light microscope (Olympus, Tokyo, Japan) to evaluate the histological changes.

### 2.13. Statistics

Using SPSS21.0, R studio, GraphPad Prism8 statistical software. The measurement data were expressed as mean ± standard deviation, and the comparison between groups was performed by two independent/paired samples students’ *t*-test, One-Way ANOVA, Two-Way ANOVA, and *p* < 0.05 was considered statistically significant.

## 3. Results

### 3.1. Time Course of Tregs Infiltrating into Brain of Mice after SAH

**Figure 1:**
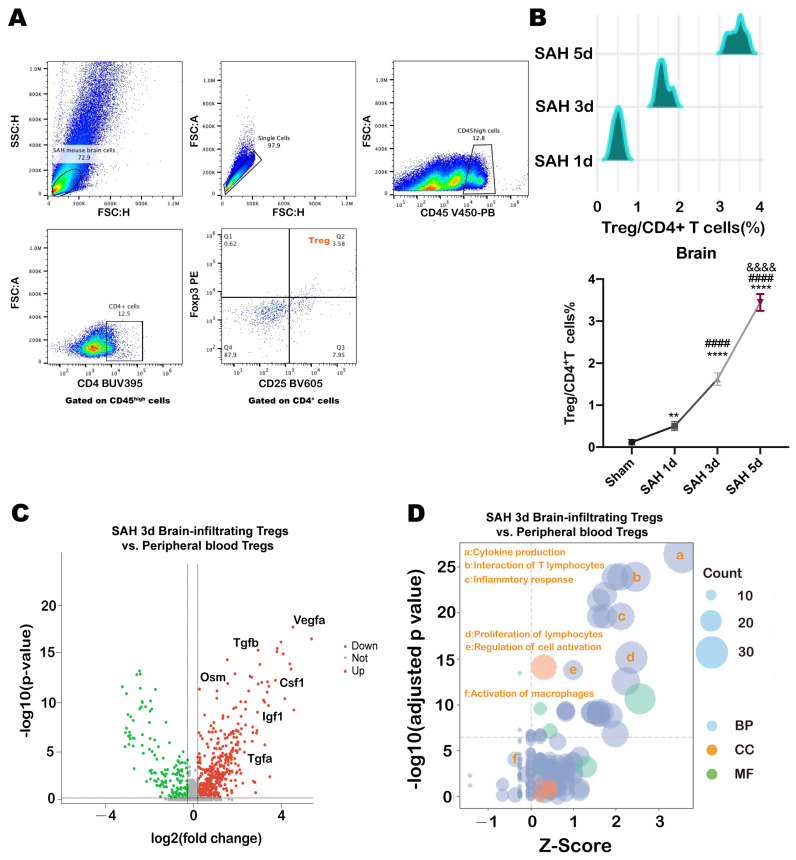
(**A**) Gating Strategies of Tregs which are infiltrating the SAH brain. (**B**) Time course of the proportion of Tregs infiltrated into SAH mice brain during 1 to 5 days after SAH induced (‘**’ vs. Sham, *p* < 0.01, ‘****’ vs. Sham, *p* < 0.0001; ‘####’ vs. SAH 1 d, *p* < 0.0001; ‘&&&&’ vs. SAH 3 d, *p* < 0.0001, One-Way ANOVA). (**C**) Volcano of gene expression variation of Brain-infiltrating Tregs vs. peripheral blood Tregs. (**D**) GO enrichment analysis of function changes of Brain-infiltrating Tregs vs. peripheral blood Tregs.

At 1, 3 and 5 days after SAH, brain tissues were taken to detect the infiltration of Tregs in the brain by flow cytometry. After gating on single cells, Tregs population were defined by CD45^high^CD4^+^CD25^+^Foxp3^+^ (Figure 1A). The infiltration of Tregs in the brain was observed within 24 h after SAH and gradually increased over time until 5 days after SAH onset. (Figure 1B).

### 3.2. Transcriptome Change of Brain-Infiltrated Tregs after SAH

The infiltrated Tregs reprogramed their transcriptome in 3 days after SAH. Compared to peripheral blood Tregs, the infiltrated Tregs upregulated the genes involved in neuroprotective factors, including Vegfa, Tgfb, Osm, Csf1, lgf1, and Tgfa (Figure 1C). Subsequently, GeneOntology(GO) enrichment analysis was performed, revealing that brain-infiltrating Tregs significantly upregulated the immunoregulation-related function, including cytokine production, interaction of T lymphocytes, inflammatory response, proliferation of lymphocytes, and regulation of cell activation (Figure 1D).

### 3.3. The Beneficial Effect of Tregs on Neurological Function in Mice after SAH

**Figure 2:**
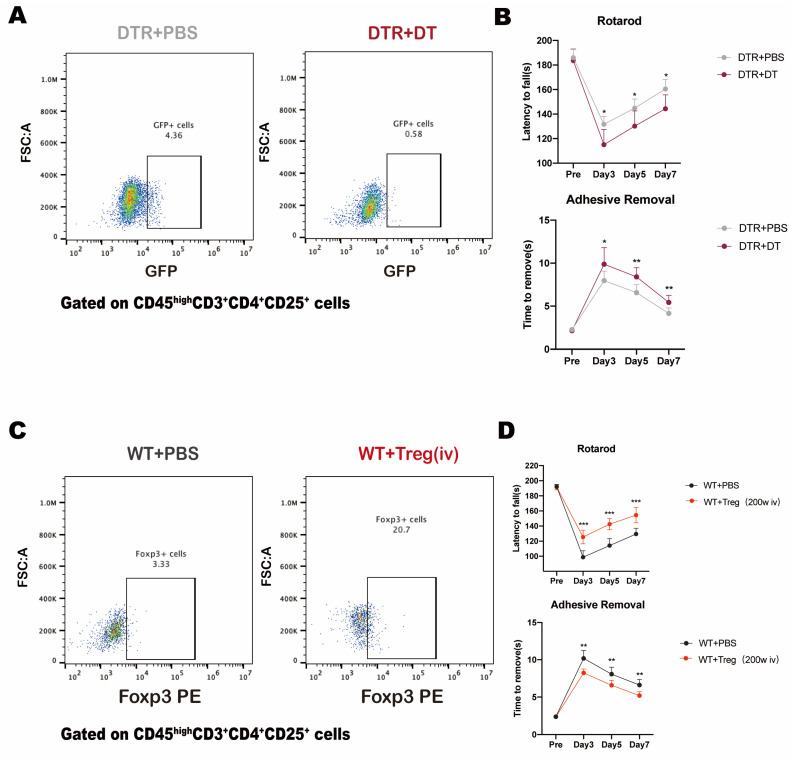
(**A**) Schematic diagram of Tregs which are infiltrating the SAH brain in the Tregs depletion group (DTR + DT) and control group (DTR + PBS). (**B**) Behavior test between Tregs depletion group (DTR+DT) and control group (DTR + PBS) (‘*’ DTR + PBS vs. DTR + DT, *p* < 0.05; ‘**’ DTR + PBS vs. DTR + DT, *p* < 0.01, One-Way ANOVA; Rotarod *p* = 0.0189; Adhesive *p* = 0.0118, Two-Way ANVOA). (**C**) Schematic diagram of Tregs which are infiltrating the SAH brain in the Tregs infusion group (WT + Treg) and control group (WT + PBS). (**D**) Behavior test between Tregs infusion group (WT + Treg) and control group (WT + PBS) (‘**’ WT + Treg vs. WT + PBS, *p* < 0.01; ‘***’ WT + Treg vs. WT + PBS, *p* < 0.001, One-Way ANOVA; Rotarod *p* = 0.0002; Adhesive *p* = 0.003, Two-Way ANVOA).

Brain-infiltrating Tregs displayed an immune-related phenotype, which might be associated with the neural repair after SAH. We then assessed neurological function after SAH on DTR mice with or without DT injection. Behavioral tests were carried out on 3, 5, 7 days after SAH induced. With DT intraperitoneal injection, the infiltration of Tregs in the brain of mice after SAH significantly decreased (Figure 2A). The neurological function of the mice in the Tregs depletion group were significantly poorer than those in the Tregs-retained group (Figure 2B).

While Tregs depletion resulted in poorer neurological function in DTR mice after SAH, we then tried to figure out whether exogenous administration of Tregs exert protective effect on WT mice after SAH. Similarly, Behavioral Tests were carried out on 3, 5, 7 days after SAH induced. After administration of exogenous Tregs, the infiltration of Tregs in the brain of mice after SAH significantly increased (Figure 2C). The neurological function of the mice in the Tregs infusion group were significantly better than those in the PBS infusion group (Figure 2D). These data suggested that brain-infiltrated Tregs exert a neuroprotective effect on the neurological function in mice after SAH.

### 3.4. Exogenous Tregs Infusion Alleviated Brain Edema and Reduced Neuron Apoptosis in Mice after SAH

**Figure 3:**
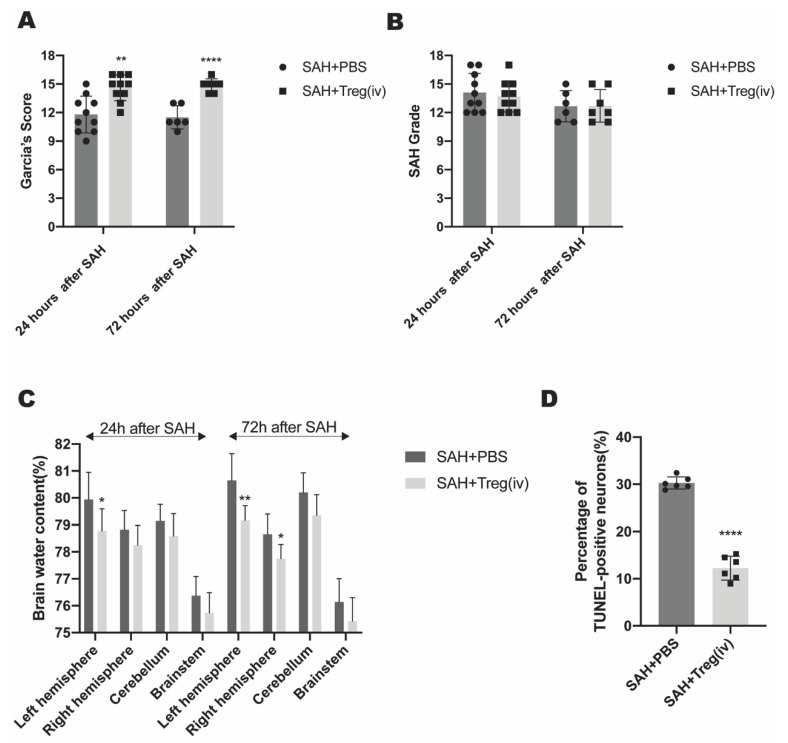
(**A**) Garcia’s Score of Tregs infusion group (SAH + Treg) and control group (SAH + PBS) mice 24 h and 72 h after SAH onset (‘**’ SAH + Treg vs. SAH + PBS, *p* < 0.01; ‘****’ SAH + Treg vs. SAH + PBS, *p* < 0.0001, students’ *t* test). (**B**) SAH Grade of Tregs infusion group (SAH + Treg) and control group (SAH + PBS) mice 24 h and 72 h after SAH onset. (**C**) Water content of different brain area of Tregs infusion group (SAH + Treg) and control group (SAH + PBS) mice 24 h and 72 h after SAH onset (‘*’ SAH + Treg vs. SAH + PBS, *p* < 0.05; ‘**’ SAH + Treg vs. SAH + PBS, *p* < 0.01, students’ *t* test). (**D**) Results of TUNEL staining of Tregs infusion group (SAH + Treg) and control group (SAH + PBS) mice brain tissue 72 h after SAH onset (‘****’ SAH + Treg vs. SAH + PBS, *p* < 0.0001, students’ *t* test).

To further verify the early neuroprotective effect of exogenous Tregs infusion after SAH. We quantified neurological score, brain edema and neuron apoptosis rate after SAH in WT mice with or without exogenous administration of Tregs. The Garcia’s Scores in the Tregs infusion group at 24 h and 72 h were significantly higher than those in the control group, suggesting that the Tregs infusion group held milder neurological deficits (Figure 3A). To avoid the deviation in SAH modeling stability, we assessed the severity of the SAH Grade. The results showed that no significant difference in the SAH Grade between the Tregs infusion group and the control group, thus ensuring the severity of SAH of two groups were relatively stable (Figure 3B). The water content in the left hemisphere of the Tregs infusion group was significantly lower than that in the control group 24 h after SAH. Furthermore, the water content of the left and right hemispheres of the Tregs infusion group was significantly lower than that of the control group 72 h after SAH, indicating that the degree of brain edema in the Tregs infusion group after SAH, comparing to the control group, was significantly improved (Figure 3C). Immunofluorescence staining showed a significantly lower proportion of TUNEL-positive cells in the Tregs infusion group than that of the control group, indicating less neuron apoptosis in the Tregs infusion group after SAH (Figure 3D). These data indicated that under the same severity of SAH, exogenous infusion of Tregs could alleviate brain edema after SAH, as well as reducing neuron apoptosis, suggesting Tregs might play a neuroprotective role in SAH mice.

### 3.5. Tregs Exerted a Neuroprotective Effect by Suppressing Neuroimmune Inflammation at Early Stage of SAH

**Figure 4:**
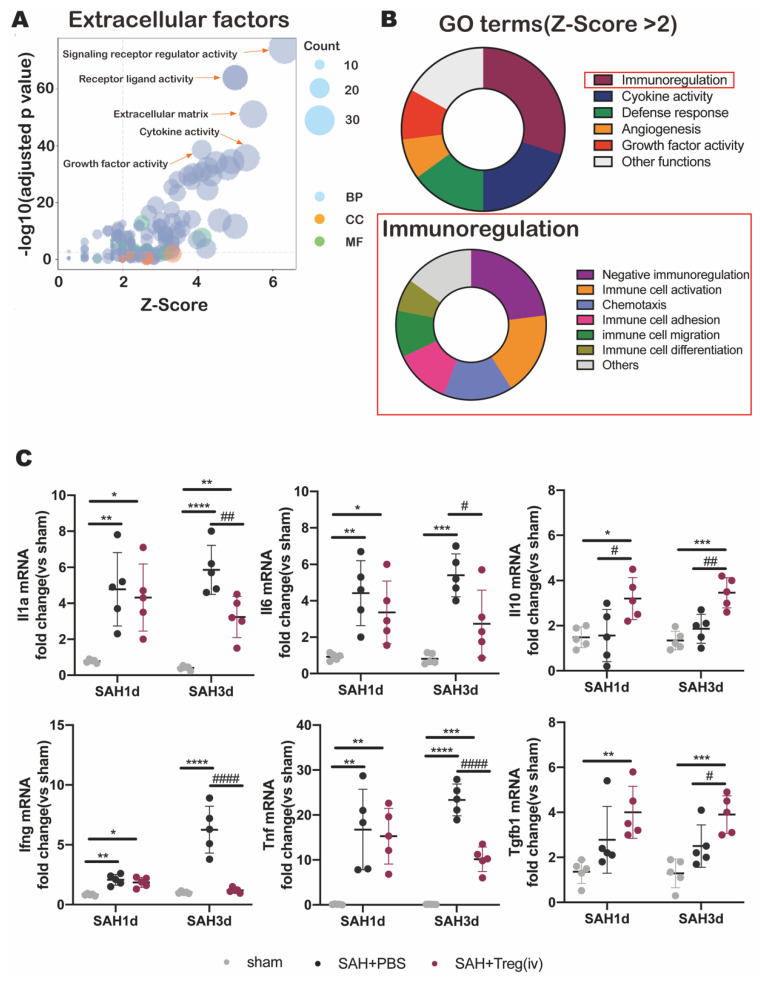
(**A**) GO enrichment of function changes in “Extracelluar factors” term of Brain-infiltrating Tregs vs. peripheral blood Tregs. (**B**) GO Terms enrichment in immune-related pathway of Brain-infiltrating Tregs vs. peripheral blood Tregs. (**C**) qPCR results of left hemisphere brain tissue after 1 day and 3 days after SAH onset of sham-operated group, SAH group and Tregs infusion group mice. (‘*’ vs. sham, *p* < 0.05, ‘**’ vs. sham, *p* < 0.01, ‘***’ vs. sham, *p* < 0.001, ‘****’ vs. sham, *p* < 0.0001; ‘#’ vs. SAH + PBS, *p* < 0.05, ‘##’ vs. SAH + PBS, *p* < 0.01, ‘####’ vs. SAH + PBS, *p* < 0.0001).

In order to further explore the specific mechanism of the neuroprotective effect of Tregs in SAH mice, we compared the changes of Tregs gene expression in the brain and peripheral blood of SAH mice. Cytokine production is an important way for immune cells to execute function. Therefore, we performed GO enrichment analysis on differential expressed genes (DEGs) related to extracellular factors. The results showed that DEGs were highly enriched in Signaling receptor modulator activity, Receptor ligand activity, Extracellular matrix, Cytokine activity and Growth factor activity (Figure 4A). Subsequently, we clustered the enriched GO terms, showing that Tregs in SAH mice brain significantly upregulated Immunoregulation-related functions, particularly in Negative immunoregulation and Immune cell activation function (Figure 4B). These data suggested that the transcriptional changes of Tregs infiltrating the brain after SAH were primarily focused on inflammation suppressive function. For the purpose of verifying the inhibition function of Tregs on neuroinflammation after SAH, we carried out qPCR to evaluate the inflammatory factors in post-SAH mice brains. The results showed that the mRNA levels of pro-inflammatory factors Il1a, Il6, Ifng, and Tnf in the Tregs infusion group were lower than those in the SAH group, while the mRNA levels of anti-inflammatory factors Il10 and Tgfb1 in the Tregs infusion group were higher than those in the SAH group (Figure 4C). These data indicated that the brain filtration of Tregs after SAH might exert a neuroprotective effect by enhancing anti-inflammatory factors expression and thereby suppressing neuroimmune inflammation.

### 3.6. Brain-Infiltrated Tregs Exert Anti-Inflammatory Effect by Secreting IL-10 after SAH

**Figure 5:**
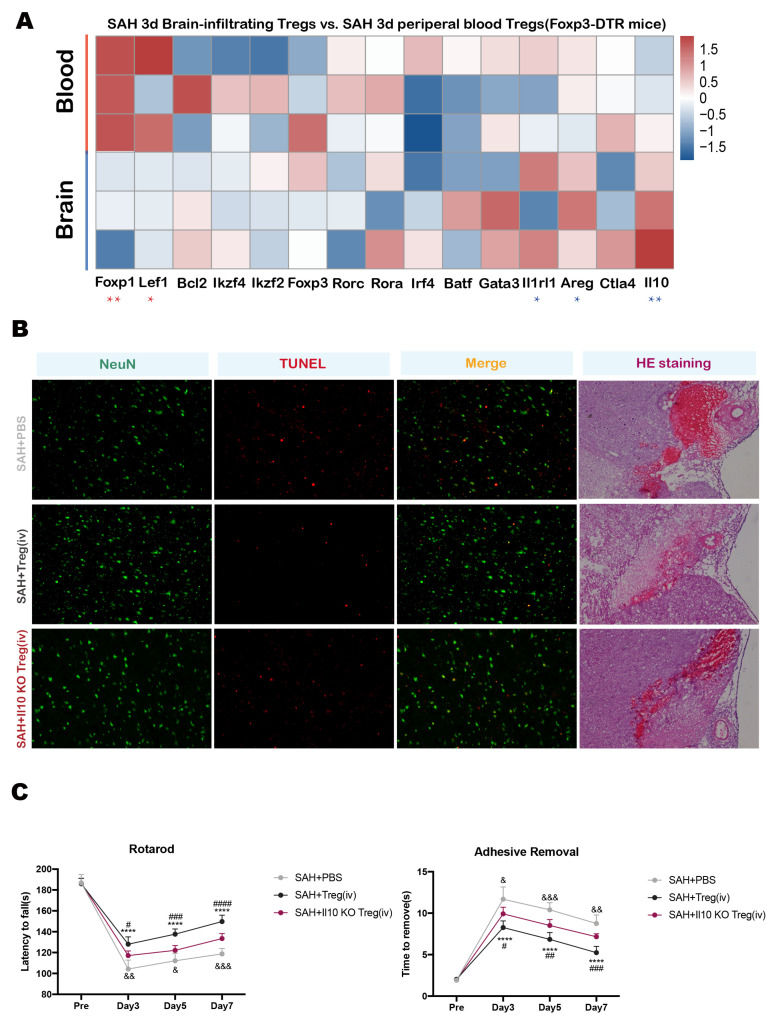
(**A**) Heatmap of transcriptome sequencing analysis of Brain-infiltrated Tregs vs. peripheral blood Tregs 3 days after SAH (Foxp3-DTR mice, ‘*’ Brain vs. Blood, *p* < 0.05, ‘**’ Brain vs. Blood, *p* < 0.01, students’ *t* test). (**B**) Immunofluorescent staining of TUNEL and NeuN and H&E staining on brain slices of 3 groups of mice (WT mice with PBS, Tregs or Il10 gene KO Tregs infusion). (**C**) Behavior test between SAH group, SAH + sham Tregs infusion group (SAH + Treg), SAH + Il10 KO Tregs infusion group (SAH + Il10 KO Treg) (WT mice with PBS, Tregs or Il10 gene KO Tregs infusion, ‘****’ SAH + Treg vs. SAH + PBS, *p* < 0.0001; ‘#’ SAH + Treg vs. SAH + Il10 KO Treg, *p* < 0.05, ‘##’ SAH + Treg vs. SAH + Il10 KO Treg, *p* < 0.01, ‘###’ SAH + Treg vs. SAH + Il10 KO Treg, *p* < 0.001, ‘####’ SAH + Treg vs. SAH + Il10 KO Treg, *p* < 0.0001; ‘&’ SAH + Il10 KO Treg vs. SAH + PBS, *p* < 0.05, ‘&&’ SAH + Il10 KO Treg vs. SAH + PBS, *p* < 0.01, ‘&&&’ SAH + Il10 KO Treg vs. SAH + PBS, *p* < 0.001, One-Way ANOVA; Rotarod *p* < 0.0001; Adhesive *p* < 0.0001, Two-Way ANOVA).

To further clarify the mechanism of Tregs which play an anti-inflammatory role in the brain, combining with GO functional enrichment results that brain-infiltrated Tregs exert immunosuppressive effect by secreting cytokines. Additionally, we sorted Tregs in the brain or peripheral blood, respectively in Foxp3-DTR mice 3 days after SAH onset for RNA sequencing and analyzed the transcriptomic changes; it was found that the Il10 gene was highly expressed in brain-infiltrated Tregs. (Figure 5A). Previous studies reported that interleukin 10 (IL-10) plays a key role in anti-inflammatory function [21]. So, as to verify whether brain-infiltrated Tregs suppress inflammatory response through IL-10. We carried out a behavioral test on 3, 5, 7 days after SAH onset in WT mice with exogenous administration of sham Treg, Il10 gene KO Treg or PBS. On the seventh day, all the mice were sacrificed and brain tissue samples were collected, and then frozen sections were made for TUNEL + NeuN staining and H&E staining to evaluate the severity of neuron apoptosis. The results showed the TUNEL-positive rate of SAH + Treg infusion group was significantly lower than that of SAH group and SAH + Il10 KO Treg infusion group (Figure 5B). Moreover, the motor and sense function of SAH + Treg infusion group were significantly better than those of the SAH + PBS group and the SAH + Il10 KO Treg infusion group, and the neurological function of the SAH + Il10 KO Treg infusion group were better than those of SAH + PBS group, but failed to reach a significant difference (Figure 5C). These data indicated that Tregs could alleviate the degree of neuron apoptosis after SAH and improve the neurological function of mice after SAH; however, these effects could be partly reversed by Il10 gene KO, suggesting that IL-10 is one of the key molecules of brain-infiltrated Tregs to exert an anti-inflammatory and neuroprotective effect.

## 4. Discussion

For decades, numerous studies suggested that delayed cerebral vasospasm is the decisive factor for the poor prognosis of SAH patients [22,23]. However, clinical trials results have shown that reducing the incidence of cerebral vasospasm did not improve the long-term neurological function of patients [24]. In recent years, early brain injury (EBI), which occurs within 72 h after SAH onset, was proposed to be an important concept affecting the acute-phase death and late neurological deficits of SAH patients [25], which includes extensive cortical depolarization [26], neuron apoptosis [27], inflammation [28], oxidative stress [29] and other pathological injury mechanisms. Among them, inflammation is considered to be one of the main reasons [30]. Humans rely on the immune system to protect the body from pathogen damage to maintain internal environment stability, rather excessive inflammatory responses may also harm the body [31]. In the early stage of brain injury after SAH, the blood and its metabolites in the subarachnoid space induce neuroinflammation, which can aggravate the degree of brain blood–barrier destruction and oxidative stress in the brain, ultimately leading to an increase of neuron apoptosis and worsening of the long-term prognosis [32]. Tregs have attracted extensive attention due to their significant immunosuppressive/immunomodulatory effects in recent years. While their role in the brain of patients with ischemic stroke has been deeply studied, the role of which in patients with SAH is not yet clear.

In this study, we used Foxp3-DTR mice and Il10 gene KO mice, employing flow cytometry, immunofluorescence staining, transcriptome sequencing and bioinformatic technology with multiple experimental designs to find out that Tregs could be detected in the brain within 24 h after SAH onset and gradually accumulated in the brain. Tregs depletion aggravated neurological deficits in mice after SAH, while exogenous infusion of Tregs improved neurological function recovery, thus, brain-infiltrated Tregs might exert neuroprotective effects. RNA sequencing analysis showed that the gene expression profiles of Tregs in the brain and blood of mice after SAH were different from those in the sham operation group, and the functional enrichment analysis of DEGs showed that Tregs upregulated anti-inflammatory function after SAH. qPCR suggested that, compared with the control group, the expression of pro-inflammatory factors of Tregs in the Treg infusion group decreased, while the expression of anti-inflammatory factors increased, indicating that Tregs suppressed immune response after SAH and played a key role in inhibiting neuroinflammation, which may be the main mechanism of its protective effect. Further downstream analysis of transcriptome sequencing data revealed that the expression of Il10 gene in brain-infiltrated Tregs was significantly upregulated. Il10 gene codes cytokine IL-10, which is one of the anti-inflammatory factors, mainly produced by monocytes and lymphocytes, and can downregulate pro-inflammatory cytokines derived from Th1 cells, NK cells, as well as macrophages [33]. Previous studies revealed that Tregs can secrete IL-10 to regulate antigen-presenting cells differentiation under inflammatory conditions, thereby inhibiting cytotoxic effector cells such as Th1 and NK cells [34,35]. In addition, it has been reported that IL-10 can alter the morphology of microglia and inhibit their release of inflammatory cytokines [36]. IL-10 reduces 50% microglia apoptosis by phosphorylation of transcription factor Stat3 [37]. After knocking out the Il10 gene, microglia mainly exhibit pro-inflammatory status and secrete a large amount of pro-inflammatory cytokines [38]. Therefore, Tregs may directly protect neurons by releasing neurotrophic factors; additionally, by releasing IL-10 to inhibit microglia-mediated inflammatory responses, Tregs may also have an indirect protective effect on neurons. We used Il10 gene KO mice and Foxp3-DTR mice to carry out a series of designed experiments, and the results showed that exogenous infusion of Tregs could alleviate the severity of neuron apoptosis after SAH and improve the neurological function of mice after SAH, which could be partly reversed after Il10 gene knockout, suggesting that IL-10 might be an important molecule for Tregs to exert their anti-inflammatory and neuroprotective effects.

Several limitations of the present study should be noted. First, Tregs suppressed neuroinflammation by various patterns and cytokines. Shi et al. reported that Tregs secreted OPN to interact with microglia and promoted white matter repair in ischemic stroke [39]. Huang et al. indicated the CD45-galectin-1 interaction between Tregs and MN9D cells prevented MPP^+^ toxicity and reduce the neuron losses in MPTP-induced Parkinson’s Disease mice with exogenous administration of Tregs [40]. In this study, we performed Bulk RNA sequencing and in vivo experiment to confirm that Tregs suppressed neuroinflammation through IL-10 in SAH. However, while mice with exogenous administration of Il10 gene KO Tregs group showed poorer neurological function than mice with sham Treg administration, the neurological function of Il10 gene KO Tregs infusion group was better than the SAH+PBS infusion group, indicating that Il10 is not the only mechanism of the protective effect of Treg. The other underlying mechanisms, such as Areg, Vegfa, Tgfa, etc., which were highly expressed in brain-infiltrating Tregs in our transcriptome data, require further studies. Second, all experiments in our study were performed in male animals, this might result in a failure to identify a potential sex bias in the phenotype and functions of Tregs. Lucia et al. reported that female Tregs were more accessible in the brain and displayed a stronger immunosuppressive effect in neonatal hypoxic-ischemic brain injury [41]. However, George et al. found that Tregs from men owned a significantly higher suppressive capacity than those from women in a clinical cross-sectional study [42]. The gender differences of Tregs and whether those differences would influence the protective effect of Tregs in SAH will be further elucidated in future studies.

## 5. Conclusions

In summary, Tregs infiltrate into the brain early after SAH, and exert a neuroprotective effect by suppressing neuroinflammation, and the cytokine IL-10 is the key to their anti-inflammatory effect. This study comprehensively reveals the role of Tregs in the early stage of brain injury after SAH, providing clinical intervention targets for the treatment of SAH in the future.

## Figures and Tables

**Table 1:** Primers for qPCR.

Gene	Forward Primer	Reverse Primer
Il1a	AAGACAAGCCTGTGTTGCTGAAGG	TCCCAGAAGAAAATGAGGTCGGTC
Il6	TCCTACCCCAACTTCCAATGCTC	TTGGATGGTCTTGGTCCTTAGCC
Il10	CCAAGCCTTATCGGAAATGA	TTTTCACAGGGGAGAAATCG
Ifng	ATGAACGCTACACACTGCATC	CCATCCTTTTGCCAGTTCCTC
Tnf	AGAAGTTCCCAAATGGCCTC	CCACTTGGTGGTTTGCTACG
Tgfb1	TGCGCTTGCAGAGATTAAAA	CGTCAAAAGACAGCCACTCA

## Data Availability

The data presented in this study are available on request from the corresponding author. The data are not publicly available due to privacy.
